# Prognostic implications of stress hyperglycemia ratio in patients with myocardial infarction with nonobstructive coronary arteries

**DOI:** 10.1080/07853890.2023.2186479

**Published:** 2023-03-10

**Authors:** Side Gao, Sizhuang Huang, Xuze Lin, Li Xu, Mengyue Yu

**Affiliations:** a Heart Center and Beijing Key Laboratory of Hypertension, Beijing Chaoyang Hospital, Capital Medical University, Beijing, China; b Department of Cardiology, Fuwai Hospital, National Center for Cardiovascular Diseases, Chinese Academy of Medical Sciences and Peking Union Medical College, Beijing, China

**Keywords:** Myocardial infarction with nonobstructive coronary arteries (MINOCA), diabetes, stress hyperglycemia ratio, cardiovascular outcomes

## Abstract

**Background:**

The role of stress hyperglycemia in acute myocardial infarction (AMI) has long been emphasized. Recently, the stress hyperglycemia ratio (SHR), a novel index reflecting an acute glycemia rise, has shown a good predictive value in AMI. However, its prognostic power in myocardial infarction with nonobstructive coronary arteries (MINOCA) remains unclear.

**Methods:**

In a prospective cohort of 1179 patients with MINOCA, relationships between SHR levels and outcomes were analyzed. SHR was defined as acute-to-chronic glycemic ratio using admission blood glucose (ABG) and glycated hemoglobin. The primary endpoint was defined as major adverse cardiovascular events (MACE), including all-cause death, nonfatal MI, stroke, revascularization, and hospitalization for unstable angina or heart failure. Survival analyses and receiver-operating characteristic (ROC) curve analyses were performed.

**Results:**

Over the median follow-up of 3.5 years, the incidence of MACE markedly increased with higher SHR tertile levels (8.1%, 14.0%, 20.5%; *p* < 0.001). At multivariable Cox analysis, elevated SHR was independently associated with an increased risk of MACE (HR 2.30, 95% CI: 1.21–4.38, *p* = 0.011). Patients with rising tertiles of SHR also had a significantly higher risk of MACE (tertile 1 as reference; tertile 2: HR 1.77, 95% CI: 1.14–2.73, *p* = 0.010; tertile 3: HR 2.64, 95% CI: 1.75–3.98, *p* < 0.001). SHR remained a robust predictor of MACE in patients with and without diabetes; whereas ABG was no longer associated with the MACE risk in diabetic patients. SHR showed an area under the curve of 0.63 for MACE prediction. By incorporating SHR to TIMI risk score, the combined model further improved the discrimination for MACE.

**Conclusions:**

The SHR independently confers the cardiovascular risk after MINOCA, and may serve as a better predictor than glycemia at admission alone, particularly in those with diabetes.

**KEY MESSAGES**
1. Stress hyperglycemia ratio (SHR) is independently associated with the prognosis in a distinct population with myocardial infarction with nonobstructive coronary arteries (MINOCA).
2. SHR is a better predictor of prognosis than admission glycemia alone, especially in diabetic patients with MINOCA.
3. SHR may serve as a prognostic marker for risk stratification as well as a potential target for tailored glucose-lowering treatment in MINOCA.

## Introduction

Acute myocardial infarction (AMI) remains the leading cause of morbidity and mortality in China and worldwide [1]. As a distinct population of AMI, patients with myocardial infarction with nonobstructive coronary arteries (MINOCA) has drawn more attention in clinical practice. As reported, MINOCA constitutes 5–10% of all AMI and they tend to be younger and more often women as compared to those with MI and obstructive coronary artery disease (MI-CAD) [2–5]. Despite the younger age and optimal secondary prevention strategies, recent studies report that patients with MINOCA are still at considerable risks of cardiovascular event both in short and long term [6–11]. Therefore, the prognosis of MINOCA is not a triviality and it is paramount to highlight the underestimated risk factors in this population.

Stress-induced hyperglycemia refers to the transient rise in glycemia during an acute illness [12]. It is quite common in AMI and has been regarded as a critical predictor of prognosis [13–17]. Previous studies often use admission blood glucose (ABG) to describe stress hyperglycemia, but an elevated value of ABG may not accurately indicate an acute glycemic rise, especially in those with chronic hyperglycemia [16–17]. Meanwhile, the combined evaluation of acute and chronic glycemia, rather than admission glycemia alone, may better identify the ‘true’ stress hyperglycemia. In 2015, Roberts et al. proposed a novel index known as stress hyperglycemia ratio (SHR) which was calculated from ABG and glycated hemoglobin (HbA_1c_) [18]. Since then, the predictive value of SHR has been verified in a variety of critical illnesses [19–21]. Recent data further confirmed that SHR could independently predict outcomes in AMI, showing a superior discrimination for adverse events than ABG alone, especially in those with diabetes [22–28].

To date, few studies have ever addressed the prognostic implications of SHR in patients with MINOCA. Here, we studied the association between SHR levels and long-term outcomes after MINOCA and analyzed whether SHR could facilitate risk prediction in this specific population.

## Methods

### Study population

**Figure 1.**
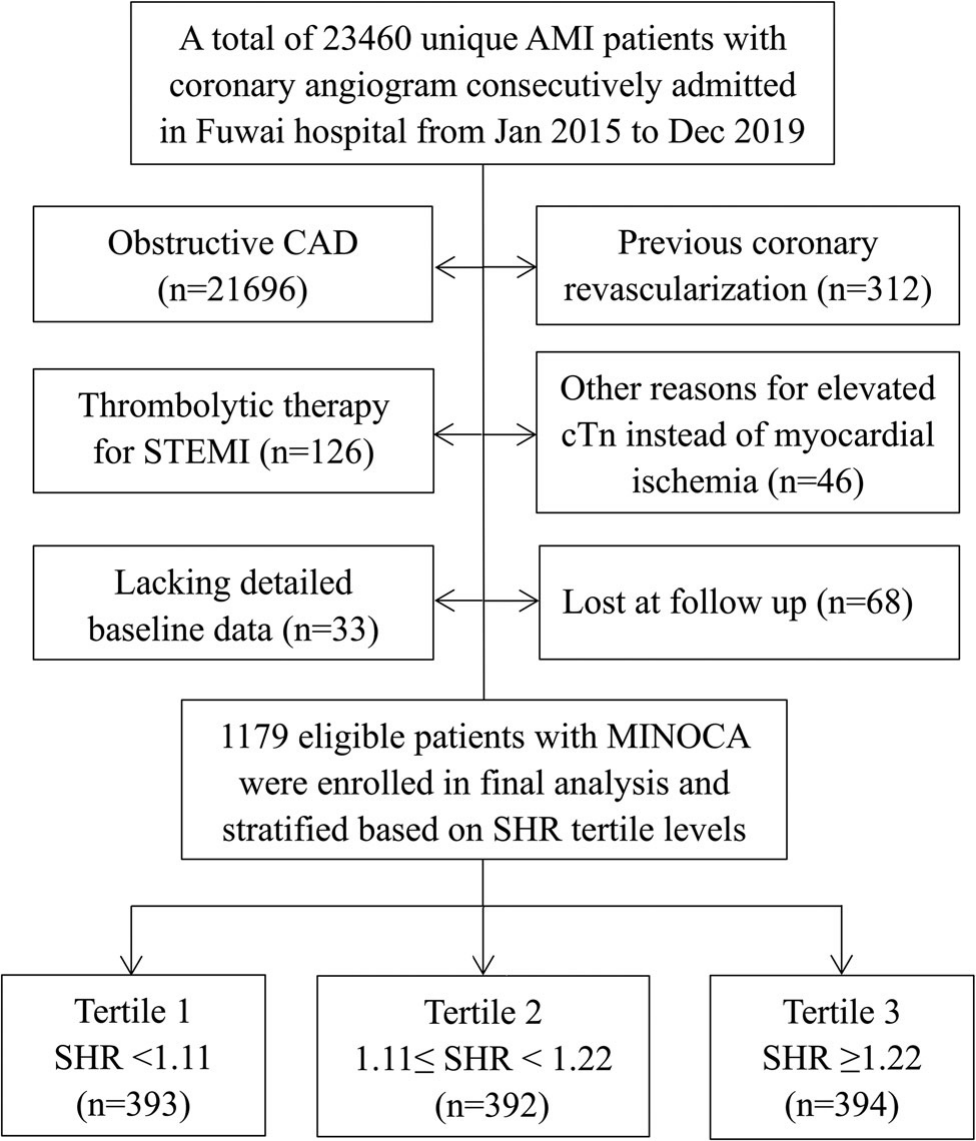
Study flowchart.

This was a single-center and prospective cohort study, which consecutively included patients with MINOCA from January 2015 to December 2019 in Fuwai hospital-the largest cardiovascular center in China. The diagnosis of MINOCA was defined based on the 4th universal definition of AMI [29] and a coronary artery stenosis of <50% proved by angiography [4,5]. As shown in Figure 1, among the hospitalized patients with AMI, the following was excluded due to: (1) obstructive coronary artery disease (CAD); (2) previous revascularization; (3) thrombolytic therapy before undergoing angiography; (4) alternate reasons for elevated troponin instead of coronary-related myocardial injury (acute heart failure, myocarditis, etc.); (5) missing data at baseline; (6) lost at follow up. Finally, a total of 1179 eligible patients presenting with MINOCA were enrolled, including ST-segment elevation myocardial infarction (STEMI) and non-ST-segment elevation myocardial infarction (NSTEMI). The evidence-based optimal medical treatments for CAD were routinely prescribed in MINOCA population, including dual anti-platelet therapy, statins, β-blocker, and angiotensin-converting enzyme inhibitor or angiotensin receptor antagonist. This study was approved by the Institutional Review Board of Fuwai hospital, and all patients provided the written informed consent to participate.

### Data collection and definition

The patients’ baseline characteristics were thoroughly reviewed and obtained from medical records. Blood samples were collected from the cubital vein for laboratory test. Blood glucose at admission (ABG) was measured upon hospital arrival using standardized biochemical assay. HbA_1c_ was routinely tested in all hospitalized patients using a liquid chromatography analyzer. The index SHR was defined as ABG divided by the estimated average chronic glucose derived from HbA_1c_ using the following formula: ABG (mmol/L)/[(1.59 ×HbA_1c_ (%)-2.59], indicating a relative glycemic increase based on recent chronic glycemia [16,17]. Serum concentrations of fasting blood glucose, lipid parameters, creatinine, high-sensitive C-reactive protein (hs-CRP), N-terminal po-B-type natriuretic peptide (NT-proBNP) at admission and peak cardiac troponin I (TnI) values were measured. The left ventricular ejection fraction (LVEF) was evaluated using the biplane Simpson method by echocardiography. The Thrombolysis in Myocardial Infarction (TIMI) risk score for NSTEMI and STEMI was calculated since admission. Diabetes mellitus (DM) was defined as a HbA_1c_ level of ≥6.5% or having a diabetic history. Hypertension was defined as repeated blood pressure ≥140/90 mmHg, past history, or taking anti-hypertensive drugs. Dyslipidemia was diagnosed by medical history or receiving lipid lowering agents.

### Endpoints and follow-up

The primary endpoint was defined as a composite of major adverse cardiovascular events (MACE) consisting of all-cause death, nonfatal MI, nonfatal stroke, revascularization, and hospitalization for unstable angina (UA) or heart failure (HF). The composite ‘hard’ endpoint included death, nonfatal MI, stroke, or revascularization. These endpoints were analyzed since admission and assessed as time to the first component event. Reinfarction was diagnosed based on the 4th universal definition of MI [29]. Revascularization was performed by the operator with discretion given the evidence of recurrent ischemia and progression of coronary lesion. Stroke was defined as neurological dysfunction and vascular brain injury caused by cerebral ischemia or hemorrhage [30]. Hospitalization for UA or HF reflected the cardiac status after AMI. Patients were regularly followed up at outpatient or *via* telephone contact at 6-month intervals by a team of independent and well-trained practitioners who were not involved in the design of this study. The endpoint events were verified and adjudicated by expert cardiologists who were blinded to the study data.

### Statistical analysis

Continuous variables were presented as mean ± standard deviation (SD) or median with interquartile range according to the normality of distribution. Differences among groups were assessed using the analysis of variance or Kruskal–Wallis *H* test. Categorical variables were expressed as numbers with percentages and were compared using Pearson’s *χ*^2^ or Fisher’s exact test. Kaplan–Meier curves were adopted to evaluate the cumulative incidence of event and the differences were analyzed by log-rank test. The univariable and multivariable Cox proportional regression analyses were used to identify the relationship between SHR levels and outcomes. Clinically relevant variables and risk factors were enrolled in the multivariate model, including age, sex, MI type (NSTEMI or STEMI), hypertension, diabetes, dyslipidemia, LVEF and peak TnI values. Hazard ratio (HR) and 95% confidence interval (CI) were calculated. Areas under the curve (AUC) were calculated to assess the accuracy of risk factors or models using receiver-operating characteristic curve (ROC) analyses. The AUC values could be interpreted as small (0.56–0.63), moderate (0.64–0.70) or strong (≥0.71) [31]. The SHR was added into the TIMI risk score to assess if the new model (SHR + TIMI) had an incremental predictive value for MACE. Accuracy of the models were compared by DeLong’s test [32]. All analyses were two-tailed and p value <0.05 was considered statistically significant. Data were analyzed using SPSS (version 22.0; SPSS Inc., IL, USA) and MedCalc Statistical Software (version 19.1; Ostend, Belgium).

## Results

### Baseline characteristics

In our cohort, men accounted for 73.5% and the mean age was 55.7 years old. The prevalence of risk factors such as hypertension and diabetes were not low. As for angiographic results, 478 patients (40.5%) had normal coronary arteries while the others (59.5%) had mild or moderate atherosclerosis. In the latter one, 466 patients had 1 vessel disease (VD), 187 had 2 VD, 48 had 3 VD, and 16 had left main disease with stenosis of <50%. Patients were divided according to the tertile levels of SHR (Figure 1), which was normally distributed in the population (Figure S1). Individuals with diabetes had much higher SHR than those without (Figure S2). As shown in Table 1, patients with higher SHR tertiles were younger. As expected, they had higher prevalence of diabetes and higher levels of admission and fasting blood glucose. They also had higher heart rate, lower LVEF, higher peak TnI, and more chance to receive emergent angiography. There were no significant differences in gender, prevalence of STEMI, hypertension, dyslipidemia, prior MI, and in-hospital medication. The BMI, blood pressures, Killip class, TIMI risk score, lipid indexes, creatinine, NT-proBNP, and hs-CRP were also similar among 3 groups. In this regard, SHR may approximately mirror a stress response after AMI, while the other baseline risk profiles were comparable across the SHR tertiles.

**Table 1.** Baseline characteristics of MINOCA patients based on SHR tertile levels.

	SHR tertile1	SHR tertile2	SHR tertile3	*p* Value
	(*n* = 393)	(*n* = 392)	(*n* = 394)	
Male, n(%)	281 (71.5%)	283 (72.1%)	303 (76.9%)	0.174
Age, yrs	57.7 ± 11.2	55.2 ± 12.2	54.0 ± 11.6	<0.001
BMI, kg/m^2^	25.7 ± 3.8	25.2 ± 3.5	25.5 ± 3.9	0.190
STEMI, *n* (%)	152 (38.6%)	153 (39.0%)	170 (43.1%)	0.364
Emergent CAG, *n* (%)	36 (9.1%)	50 (12.7%)	73 (18.5%)	0.001
Vital signs at admission				
Systolic BP, mmHg	125.1 ± 16.3	125.5 ± 18.6	125.2 ± 17.6	0.943
Diastolic BP, mmHg	75.4 ± 11.0	76.4 ± 11.5	77.4 ± 12.6	0.065
Heart rate, bpm	67.6 ± 9.8	69.2 ± 10.1	71.5 ± 12.2	<0.001
Medical history				
Hypertension	207 (52.6%)	198 (50.5%)	225 (57.1%)	0.151
Diabetes	58 (14.7%)	71 (18.1%)	107 (27.1%)	<0.001
Dyslipidemia	224 (56.9%)	217 (55.3%)	245 (62.1%)	0.113
Previous MI	16 (4.0%)	21 (5.3%)	21 (5.3%)	0.635
Killip class ≥ 2, *n* (%)	26 (6.6%)	31 (7.9%)	32 (8.1%)	0.379
LVEF, %	61.2 ± 6.4	61.1 ± 6.5	59.2 ± 9.0	<0.001
TIMI risk score	3.2 ± 1.1	3.3 ± 1.2	3.5 ± 1.4	0.531
Laboratory data				
SHR	1.02 ± 0.08	1.16 ± 0.03	1.36 ± 0.16	<0.001
ABG, mmol/L	7.66 ± 1.45	7.86 ± 1.49	8.54 ± 2.16	<0.001
FBG, mmol/L	5.35 ± 1.40	5.55 ± 1.43	6.20 ± 2.07	<0.001
HbA_1c_, %	5.88 ± 0.92	5.87 ± 0.96	6.08 ± 1.03	0.063
TG, mmol/L	1.47 (1.04, 2.00)	1.41 (1.08, 1.89)	1.50 (1.03, 2.07)	0.659
TC, mmol/L	3.86 ± 0.88	3.96 ± 0.89	3.94 ± 0.94	0.250
LDL-C, mmol/L	2.25 ± 0.74	2.34 ± 0.77	2.27 ± 0.77	0.257
HDL-C, mmol/L	1.06 ± 0.29	1.08 ± 0.29	1.10 ± 0.29	0.155
Creatinine, μmol/L	80.6 ± 18.8	79.2 ± 15.6	80.4 ± 19.0	0.519
NT-proBNP, pg/mL	363 (102, 676)	375 (115, 681)	382 (126, 689)	0.198
Peak TnI, ng/mL	3.13 (0.65, 5.93)	3.22 (0.72, 6.52)	3.45 (0.81, 6.93)	0.033
hs-CRP, mg/L	2.19 (1.03, 5.68)	2.25 (1.01–5.26)	2.23 (1.05, 6.64)	0.351
In-hospital medication				
DAPT	367 (93.3%)	368 (93.8%)	356 (90.3%)	0.126
Statin	373 (94.9%)	376 (95.9%)	381 (96.7%)	0.452
ACEI or ARB	269 (68.4%)	250 (63.7%)	240 (60.9%)	0.084
Beta-blocker	287 (73.0%)	288 (73.4%)	285 (72.3%)	0.937
CV outcomes				
MACE	32 (8.1%)	55 (14.0%)	81 (20.5%)	<0.001
Death, MI, stroke or revascularization	24 (6.1%)	33 (8.4%)	45 (11.4%)	0.029
All-cause death	3 (0.7%)	5 (1.2%)	10 (2.5%)	0.111
Nonfatal MI	9 (2.2%)	14 (3.5%)	18 (4.5%)	0.124
Revascularization	10 (2.5%)	13 (3.3%)	23 (5.8%)	0.043
Nonfatal stroke	4 (1.0%)	3 (0.7%)	5 (1.2%)	0.777
Hospitalization for UA	14 (3.5%)	23 (5.8%)	34 (8.6%)	0.011
Hospitalization for HF	10 (2.5%)	17 (4.3%)	21 (5.3%)	0.133

Patients were divided according to tertile levels of SHR (Tertile1: SHR <1.11, Tertile2: 1.11≤ SHR <1.22, Tertile3: SHR ≥1.22). SHR was calculated using the formula of ABG (mmol/L)/ [(1.59 ×HbA_1c_ (%) – 2.59]. BMI: body mass index; STEMI: ST-segment elevation myocardial infarction; CAG: coronary angiography; BP: blood pressure; LVEF: left ventricular ejection fraction; TIMI: Thrombolysis in Myocardial Infarction; SHR: stress hyperglycemia ratio; ABG: admission blood glucose; FBG: fasting blood glucose; HbA_1c_: glycated hemoglobin; TG: triglyceride; TC: total cholesterol; LDL-C: low-density lipoprotein cholesterol; HDL-C: high-density lipoprotein cholesterol; NT-proBNP: N-terminal pro-B-type natriuretic peptide; TnI: Troponin I; hs-CRP: high-sensitive C-reactive protein; DAPT: dual anti-platelet therapy; ACEI: angiotensin-converting enzyme inhibitor; ARB: angiotensin receptor antagonist; MACE: major adverse cardiovascular events; MI: myocardial infarction; UA: unstable angina; HF: heart failure.

### Association between SHR and outcomes

**Figure 2.**
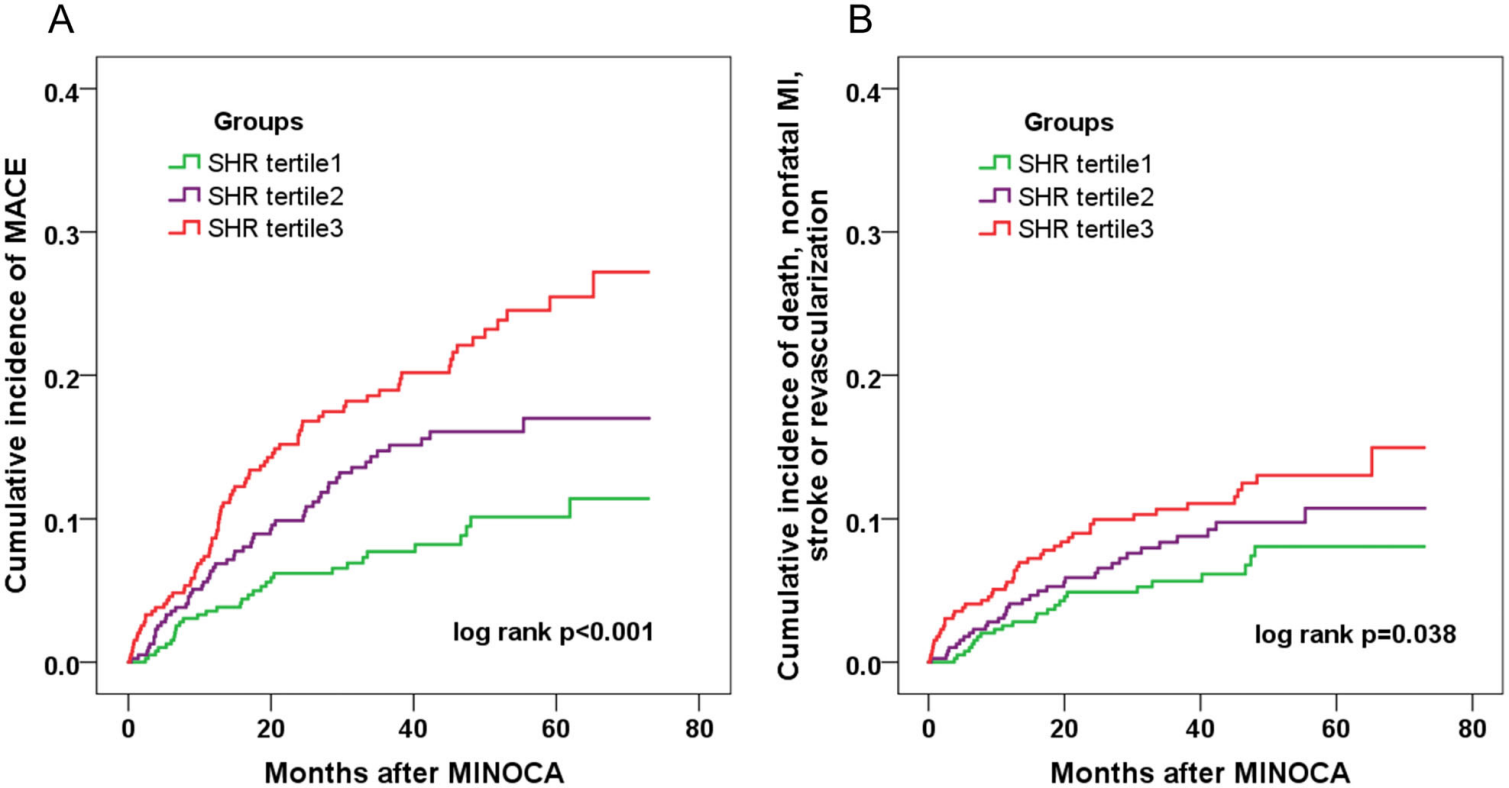
Incidence of the composite event in MINOCA patients across the SHR tertiles. Kaplan–Meier curves showing the cumulative incidence of MACE (A), and the composite hard endpoint of death, nonfatal MI, stroke, or revascularization (B) in patients with tertiles of SHR.

A total of 168 patients developed MACE during the median follow-up of 3.5 years. Of these, 18 died, 41 suffered reinfarction, 12 had stroke, 46 had revascularization, 71 was hospitalized for UA and 48 for HF. Patients with rising tertiles of SHR had a significantly higher incidence of MACE (8.1, 14.0, 20.5%; *p* < 0.001) and the composite hard endpoint of death, reinfarction, revascularization or stroke (6.1, 8.4, 11.4%; *p* = 0.029) (Table 1). The Kaplan–Meier curves also exhibited a higher cumulative incidence of MACE and the composite hard endpoint in higher SHR tertile groups (Figure 2). As for each single endpoint, however, there were no significant differences in the risk of death, MI, or stroke except for revascularization and hospitalization for UA.

**Figure 3.**
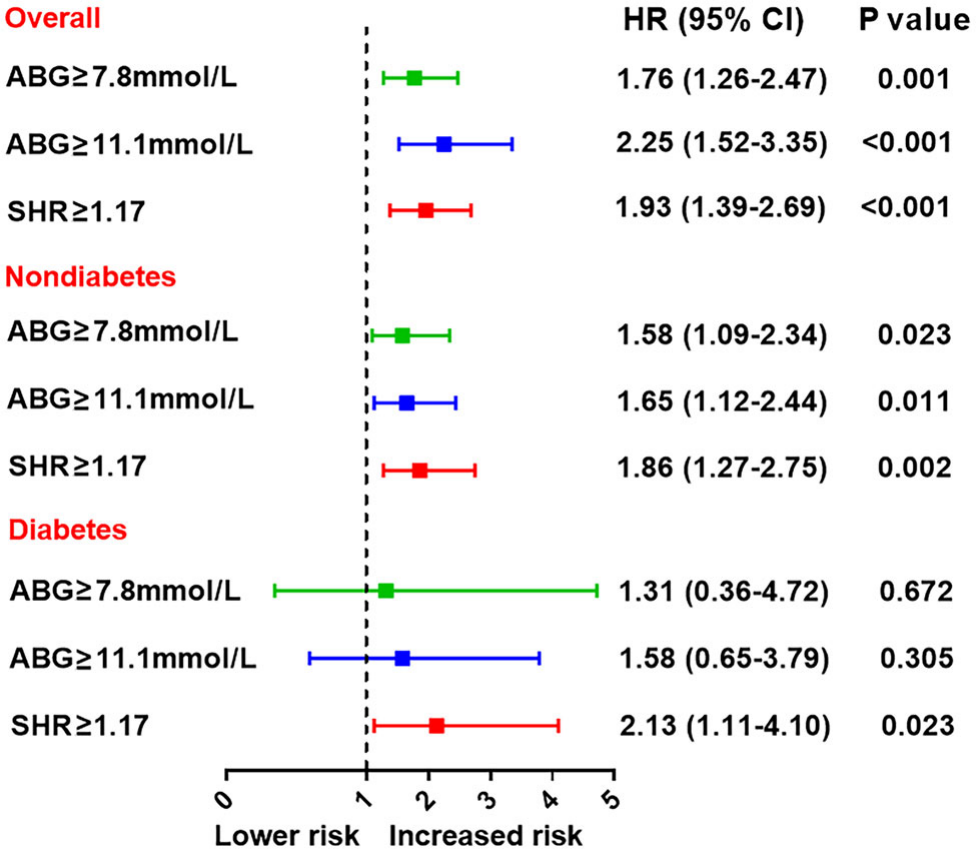
Prognostic value of the ABG or SHR-defined stress hyperglycemia for MACE. Effect of stress hyperglycemia defined by ABG or SHR on MACE risk in overall and in patients with or without diabetes. Hyperglycemia was defined as ABG ≥ 7.8mmol/L, ABG ≥ 11.1mmol/L, or SHR ≥ 1.17. The two cutoff values of ABG were adopted in line with current practice while the cutoff value of SHR was identified with the maximum Youden index for MACE prediction via ROC analysis. HR was adjusted for age, sex, MI type, hypertension, dyslipidemia, LVEF and peak TnI values at multivariable Cox analysis. ABG: admission blood glucose; SHR: stress hyperglycemia ratio; HR: hazard ratio; CI: confidence interval.

At multivariable Cox analysis, elevated SHR was significantly associated with an increased risk of MACE, even after adjustment for major confounders (for per 1SD increase in SHR, HR 2.30, 95% CI: 1.21–4.38, *p* = 0.011). The adjusted risk of MACE also increased with rising SHR tertiles (tertile 1 as reference; tertile 2: HR 1.77, 95% CI: 1.14–2.73, *p* = 0.010; tertile 3: HR 2.64, 95% CI: 1.75–3.98, *p* < 0.001) (Table 2). SHR remained an independent predictor of MACE in subgroups of diabetes or non-diabetes whereas ABG was no longer associated with MACE risk in diabetic patients (Table 3). The cutoff value of SHR was identified as 1.17 via ROC analysis that maximized the sensitivity and specificity for MACE prediction. The prognostic values of ABG- and SHR-defined hyperglycemia (SHR ≥1.17, ABG ≥7.8 mmol/L or ABG ≥11.1 mmol/L) were further compared (Figure 3). Both ABG and SHR-defined hyperglycemia performed well in predicting MACE in nondiabetic patients; yet, only the latter one was significantly correlated with the MACE risk in diabetic patients, suggesting that SHR may serve as a better predictor of MACE than ABG alone.

**Table 2.** Association between SHR levels and the event risk.

Groups	Unadjusted Cox analysis		Adjusted Cox analysis	
	HR (95% CI)	*p* Value	HR (95% CI)	*p* Value
MACE				
SHR, per 1SD increase	2.73 (1.43–5.20)	0.002	2.30 (1.21–4.38)	0.011
SHR tertile1	1 (Reference)	…	1 (Reference)	…
SHR tertile2	2.10 (1.35–3.28)	0.001	1.77 (1.14–2.73)	0.010
SHR tertile3	3.06 (2.01–4.65)	<0.001	2.64 (1.75–3.98)	<0.001
Death, nonfatal MI, stroke or revascularization				
SHR, per 1SD increase	2.80 (1.28–6.16)	0.010	2.33 (1.07–5.08)	0.033
SHR tertile1	1 (reference)	…	1 (reference)	…
SHR tertile2	1.69 (1.01–2.89)	0.042	1.50 (0.88–2.55)	0.130
SHR tertile3	2.04 (1.23–3.37)	0.005	1.88 (1.14–3.08)	0.012

Association of SHR as a continuous variable (for per 1SD increase) and a categorical variable (tertile1 as reference) with the event risk. HR was adjusted for age, sex, MI type (NSTEMI or STEMI), hypertension, diabetes, dyslipidemia, LVEF and peak TnI in the multivariate model. HR: hazard ratio; CI: confidence interval; SD: standard deviation; SHR: stress hyperglycemia ratio; MACE: major adverse cardiovascular events; MI: myocardial infarction.

**Table 3.** SHR versus ABG for MACE prediction in patients with or without diabetes.

Variables	Unadjusted Cox analysis		Adjusted Cox analysis	
	HR (95% CI)	*p* Value	HR (95% CI)	*p* Value
Overall				
ABG	1.09 (1.02–1.16)	0.013	1.08 (1.01–1.16)	0.025
SHR	2.73 (1.43–5.20)	0.002	2.30 (1.21–4.38)	0.011
Non-diabetes				
ABG	1.29 (1.04–1.59)	0.017	1.26 (1.02–1.56)	0.029
SHR	3.04 (1.28–7.19)	0.011	2.78 (1.26–6.12)	0.016
Diabetes				
ABG	1.04 (0.92–1.16)	0.511	1.02 (0.91–1.15)	0.665
SHR	3.76 (1.23–11.41)	0.019	3.52 (1.14–10.84)	0.028

Performance of SHR versus ABG for MACE prediction. The multivariate model included age, sex, MI type, hypertension, diabetes (excluded in DM and non-DM subgroups), dyslipidemia, LVEF and peak TnI value. HR for per 1 SD increased in ABG or SHR. HR: hazard ratio; CI: confidence interval; SD: standard deviation; SHR: stress hyperglycemia ratio; ABG: admission blood glucose; AUC: areas under the curve.

### Predictive value of SHR for MACE

**Figure 4.**
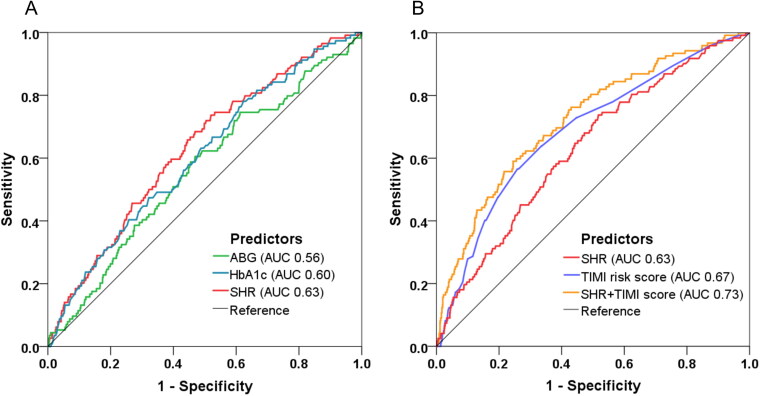
Model improvement in predicting MACE. Receiver operating characteristic (ROC) curves showing discriminatory ability of SHR, FBG, ABG, HbA1c for MACE (A) and the combined model incorporating SHR and TIMI risk score for MACE prediction (B). SHR: stress hyperglycemia ratio; ABG: admission blood glucose; FBG: fasting blood glucose; HbA_1c_: glycated hemoglobin; TIMI: thrombolysis in myocardial infarction; AUC: area under the curve.

At ROC analysis, SHR yielded a better predictive value of MACE (AUC 0.63, 95% CI: 0.57–0.68, *p* < 0.001) than ABG or HbA_1c_ alone (Figure 4(A)). Beyond the TIMI risk score (AUC 0.67, 95% CI: 0.62–0.72, *p* < 0.001), the incorporation of SHR into TIMI risk score further improved the discrimination of MACE and thus enabled a more accurate risk prediction (AUC from 0.67 to 0.73, ΔAUC 0.06, *p* = 0.017 by DeLong’s test) (Figure 4(B)).

## Discussion

The present study, for the first time, confirmed the prognostic power of SHR in patients with MINOCA. This index combined the evaluation of acute and chronic glycemia and may better describe stress hyperglycemia. As compared with admission glycemia alone, SHR had a better predictive value of MACE, especially in diabetic patients. SHR further provided incremental model improvement in MACE prediction on the basis of an established risk score. These data support the utility of SHR as a biomarker for post-MI risk stratification in MINOCA patients.

MINOCA represents a distinct clinical entity and the underlying mechanisms may include plaque disruption, thromboembolism, coronary spasm, dissection, microvascular dysfunction and supply-demand mismatch [2]. Although this population are younger and have no obstructive coronary arteries, they seem to have similar cardiovascular risk profiles compared to those with MI-CAD [3] and their prognosis has been increasingly concerned. Previous research have shown a considerably high risk of mortality and adverse events in patients with MINOCA [6–11]. Some studies even reported a similar prognosis between MINOCA and MI-CAD population despite the optimal strategies for secondary prevention [6,7]. Similarly, we found that the clinical course of MINOCA in our cohort was not as benign as expected. Approximately 1.5% of patients died and 14.2% of them experience MACE during the follow-up. This alarming fact should remind us physicians to be alert and take more aggressive efforts in targeting residual risk factors and improving outcomes for this population.

Stress hyperglycemia emphasizes a relative acute increase of glycemia in response to stress reaction or critical illness [12]. It is commonly seen in AMI and has been reported as a powerful predictor of worse outcomes both in diabetic and nondiabetic patients with AMI [13–17]. Previous studies have revealed that stress hyperglycemia can activate the neuroendocrine system, release excessive catecholamine and cytokines, aggravate inflammatory response and oxidative stress, promote a prothrombotic state, induce endothelial dysfunction, and impair microcirculatory function [33–37]. In line with these pathophysiologic changes, patients with stress hyperglycemia tend to have larger infarct size, worse cardiac function, and a higher risk of plaque progression, heart failure, ventricular arrhythmia, and death after AMI [13–17]. Thus, it is critical to adequately evaluate stress hyperglycemia for early risk stratification and pre-emptive decision-making.

Glycemia at admission has long been used to evaluate stress hyperglycemia, but it is simply a glucose level of point-in-time and may not reflect the status of acute glycemic fluctuation. It is no doubt that AMI patients with diabetes have a worse outcome than those without. However, emerging data show that elevated glucose at admission is a powerful predictor in non-diabetes, but it may be not a robust risk factor in diabetes [23–25]. This indicates that the deleterious effect of acute hyperglycemia is more pronounced in nondiabetic patients and may be blunted in those with diabetes. In this regard, an acute-to-chronic glycemic ratio may better describe the stress-induced violent fluctuations in glycemia rather than admission glycemia alone. Following this assumption, a novel metric known as SHR has been firstly introduced by Roberts et al. [18]. In this index, acute glycemia is shown as ABG and chronic glycemia is estimated by HbA_1c_. It is reported that SHR reflects the magnitude of a relative glycemic rise and is a better predictor of morbidity and mortality in critical illness across the whole glycemic spectrum [18]. Recently, the prognostic value of SHR has been confirmed in different subpopulations with AMI [24–28]. Patients with higher SHR had worse prognosis after AMI, and SHR was superior to admission glycemia alone as for risk prediction, especially in those with diabetes [23–25].

Consistent with previous data, our study has extended the implication of SHR to MINOCA patients. In our cohort, the adjusted risk of MACE markedly increased with higher SHR tertiles. Both ABG and SHR performed well for MACE prediction in nondiabetic patients, while only SHR remained a robust predictor in diabetic ones. Stress hyperglycemia defined by SHR ≥ 1.17 also showed a significant discrimination of MACE, especially in diabetic patients. When added to a traditional risk score, elevated SHR still exhibited incremental prognostic power. However, apart from the combined outcomes, the hard endpoint such as death or reinfarction actually did not differ significantly across SHR tertiles. We note that the event numbers are small and may not be efficient for statistical significance. So, our findings need to be verified by future studies.

In clinical practice, the optimal treatment for stress hyperglycemia remains a dilemma. Some studies found that a tight glycemic control strategy in AMI failed to yield an improved outcome. It is reported that insulin-base therapy would not reduce mortality after an AMI [38,39]. A neutral or even deleterious effect of intensive glucose-lowering therapy was also confirmed in diabetic patients [40,41]. Indeed, the risk of hypoglycemia induced by insulin can result in acute glycemic variability and impose a harmful impact on prognosis. However, recent data showed that a strict glycemic control during AMI could reduce inflammatory responses and increase regenerative potential of myocardium [42,43]. The use of GLP-1 receptor agonist and SGLT-2 inhibitors can exhibit cardioprotective effects without the risk of hypoglycemia even in acute condition such as AMI [44], thus making the intensified management of glucose more beneficial. In this aspect, the index SHR can not only serve as a prognostic marker, but also a potential therapeutic target for tailored treatment. For those who had glycemia below the conventional treatment threshold of 11 mmol/L, SHR may help to discriminate a real glycemic rise and assist physicians to decide when to initiate the glucose-lowering therapy. Still, we should note that the prognostic value of SHR is moderate and far from claiming perfection. Its implications in MINOCA need further validation, and the effectiveness of SHR-targeted therapy in AMI also warrants more research.

## Limitation

There were several limitations in our study. First, this cohort was derived from a single center. The sample size is limited and the event numbers are small, especially for hard endpoints. This may not reach the required number of statistical significance, and thus the event risk (such as death) did not differ significantly among groups. Hence, our data are needed to be validated by future larger-scale studies or nationwide registries which can be more representative. Second, this was an observational study. Residual confounding factors and selection bias may exist and influence the results despite multivariable adjustment and subgroup analyses. Thus, we cannot conclude a causal impact of SHR on outcomes in MINOCA, which warrants further research. Third, coronary optical coherence tomography (OCT) and cardiac magnetic resonance imaging (MRI) is useful to determine underlying causes of MINOCA. However, we did not perform the multi-modality imaging for everyone (only 51 patients had OCT and 62 had MRI). Thus, the value of SHR in different phenotypes of MINOCA is unknown. Fourth, the acute glucose levels can fluctuate over time. Yet, we cannot standardize the time and circadian patterns in which the ABG levels were measured. Besides, SHR was only assessed at baseline. Its dynamic changes and fluctuation pattern during treatment and follow-up were not recorded, which might provide more clinical implications.

## Conclusions

Elevated SHR was closely associated with an increased risk of MACE in MINOCA patients. As a valid index of stress hyperglycemia, SHR performed better in risk prediction as compared with glycemia at admission alone, especially in diabetic patients. Routine assessment of SHR may help to identify high-risk patients and facilitate the tailored glucose-lowering therapies.

## Supplementary Material

Supplemental Material

## Data Availability

The present study was approved by the Ethics Committee of Fuwai hospital (Approval No. 2012-431). Datasets used or analyzed during the current study are available from the corresponding author on reasonable request.
