# Supplementary material for: Prognostic implications of stress hyperglycemia ratio in patients with myocardial infarction with nonobstructive coronary arteries
Source: Ann Med. 2023 Mar 10;55(1):990–9. doi: 10.1080/07853890.2023.2186479 (PMC10795641; doi:10.1080/07853890.2023.2186479)
Supplement: Supplemental Material [file IANN_A_2186479_SM1462.docx]

**Supplementary Material of the Manuscript**


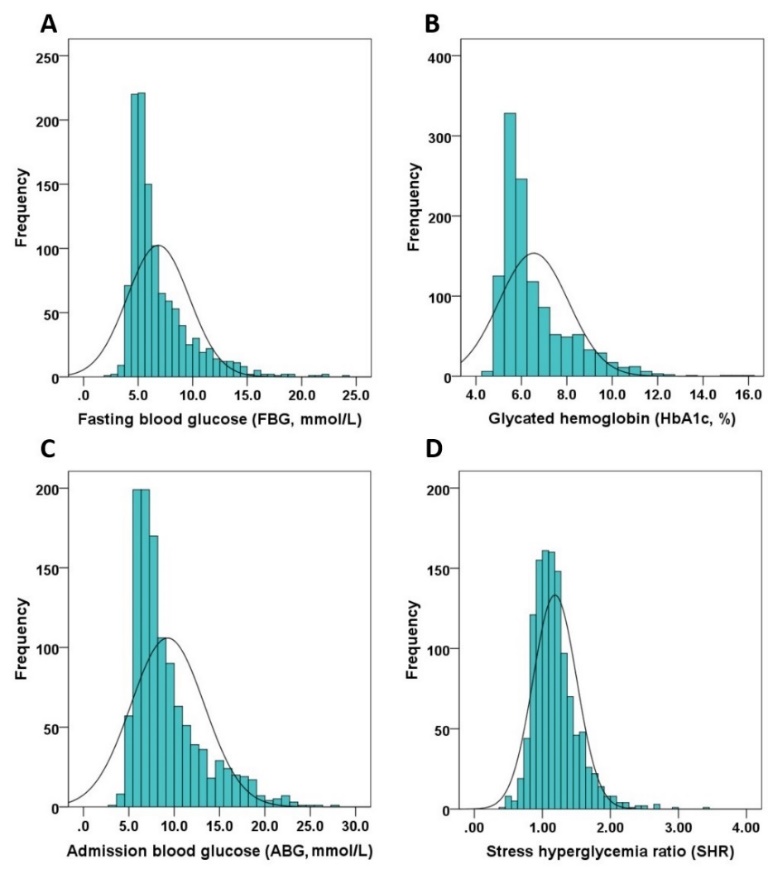


**Figure S1. Distribution of FBG, HbA_1c_, ABG and SHR in patients.**

Distribution of fasting blood glucose (A), glycated hemoglobin (B), admission blood glucose (C), and stress hyperglycemia ratio (D) in all patients.

**Figure S2.** **Levels of SHR in patients** **with different glucometabolic status.**
